# Determination of T Follicular Helper Cell Fate by Dendritic Cells

**DOI:** 10.3389/fimmu.2018.02169

**Published:** 2018-09-27

**Authors:** Jayendra Kumar Krishnaswamy, Samuel Alsén, Ulf Yrlid, Stephanie C. Eisenbarth, Adam Williams

**Affiliations:** ^1^Bioscience, Respiratory, Inflammation and Autoimmunity, IMED Biotech Unit, AstraZeneca, Gothenburg, Sweden; ^2^Department of Microbiology and Immunology, Institute of Biomedicine, Sahlgrenska Academy, University of Gothenburg, Gothenburg, Sweden; ^3^Department of Laboratory Medicine, Yale University School of Medicine, New Haven, CT, United States; ^4^Department of Immunobiology, Yale University School of Medicine, New Haven, CT, United States; ^5^The Jackson Laboratory for Genomic Medicine, Farmington, CT, United States; ^6^Department of Genetics and Genomic Sciences, University of Connecticut Health Center, Farmington, CT, United States

**Keywords:** dendritic cell, Tfh cell, DC subset, DC migration, humoral response, vaccine

## Abstract

T follicular helper (Tfh) cells are a specialized subset of CD4^+^ T cells that collaborate with B cells to promote and regulate humoral responses. Unlike other CD4^+^ effector lineages, Tfh cells require interactions with both dendritic cells (DCs) and B cells to complete their differentiation. While numerous studies have assessed the potential of different DC subsets to support Tfh priming, the conclusions of these studies depend heavily on the model and method of immunization used. We propose that the location of different DC subsets within the lymph node (LN) and their access to antigen determine their potency in Tfh priming. Finally, we provide a three-step model that accounts for the ability of multiple DC subsets and related lineages to support the Tfh differentiation program.

## Tfh cells in immunity

It was initially believed that B cell activation and antibody production were regulated by the Th2 CD4^+^ T cell effector subset. Yet a principal role of effector T cells in the immune response is to deal with pathogens or tissue damage, most often outside of secondary lymphoid organs (SLOs). Indeed, upon activation, effector T cells rapidly downregulate homing receptors that keep them in the lymph node, thereby enabling migration to affected tissues. It was therefore unclear how CD4^+^ T cell help for B cells in the follicles could occur until the discovery of T follicular helper (Tfh) cells. Tfh cells are a subset of CD4^+^ T cells that function in the lymph node (LN) and spleen to promote survival, affinity maturation, and class switch recombination of B cells (1, 2). Tfh cells express high levels of a cell cycle inhibitor called programmed cell death-1 (PD-1), inducible T cell co-stimulator (ICOS) and the chemokine receptor CXCR5. CXCR5 expression localizes Tfh cells to B cell-rich areas of SLOs. These markers, in combination with the lineage-defining transcription factor BCL6, allow identification of the Tfh subset by flow cytometry [for review see (3)]. Discovery and characterization of the Tfh subset has illuminated the pathology underlying numerous diseases such as lupus as well as providing a clearer understanding of a primary cellular regulator of effective vaccine responses (4).

## Stages of Tfh induction

How are Tfh cells induced during an immune response? It is clear that dendritic cells (DCs) and B cells must cooperate to induce and then solidify the Tfh fate (5–9). The DC phase occurs over the first couple of days following T cell recognition of cognate antigen and induces a “pre-Tfh” state. In the absence of further interactions with an activated B cell, these nascent Tfh cells dissipate (10, 11). Instead, if the B cell phase ensues, a “committed” Tfh cell is produced that has the ability to enter the germinal center (GC) and in turn promote B cell proliferation, class switching, and affinity maturation. Using murine immunization models that provide high concentrations of antigen together with an adjuvant, the first DC phase can be bypassed by B cells or monocytes and plasmacytoid DCs (pDCs) (12). However, under most immunization conditions, MHCII-expressing DCs are both necessary and sufficient to induce pre-Tfh cells (6). As we will review, recent work has illuminated the nature of the DC subset capable of this step during particular types of immunizations. In both mouse and man, Tfh cells can be divided into additional subsets based on their differential expression of cytokines and chemokine receptors (13, 14). These Tfh subsets have been proposed to promote particular antibody isotypes from B cells. For example, Tfh1 cells express IFNγ, are produced during a type 1 immune response and can direct IgG2 class switching (13). DCs may also play an important role in the polarization of particular Tfh subsets. Indeed, Pattarini et al. recently showed that human Thymic Stromal Lymphopoietin (TSLP)-activated DCs seem to favor the polarization of naïve T cells into Tfh2 cells (15). However, how the different Tfh fates are induced and the particular role of DCs in guiding differentiation remains to be fully elucidated and is an area of active research.

## Dendritic cell subsets

**Figure 1:**
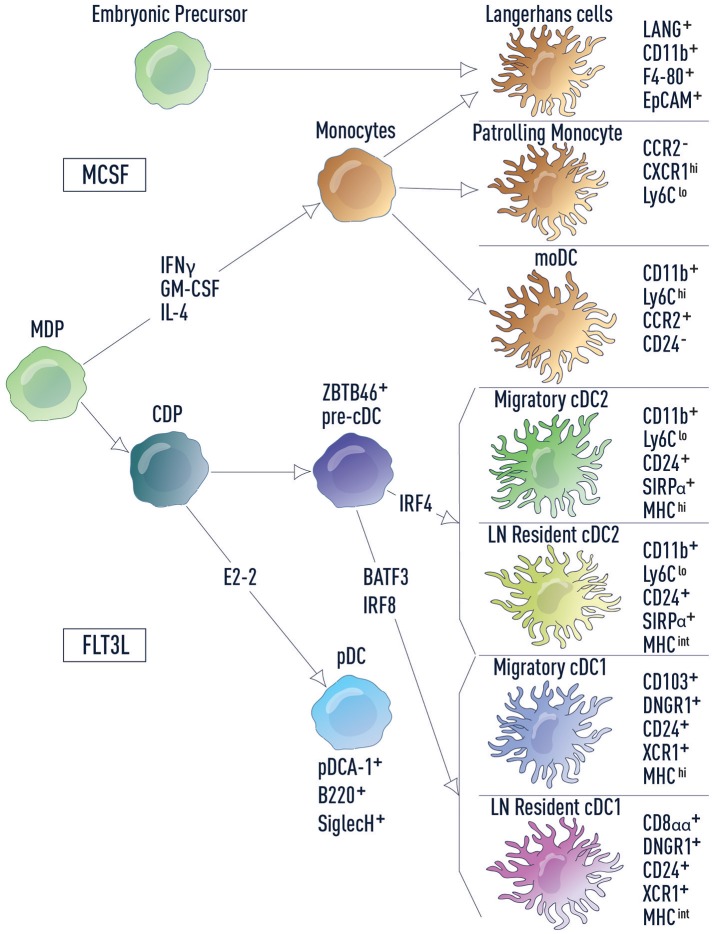
DC subsets and related lineages. DC subsets and related lineages can be annotated based on ontogeny and function. Monocyte-macrophage DC progenitors (MDPs) differentiate into monocytes and common dendritic cell progenitors (CDPs). Monocytes further differentiate into patrolling monocytes, inflammatory monocytes (including moDCs) and Langerhans cells. Langerhans cells also develop from embryonic precursors. CDPs in turn give rise to both pDCs and pre-cDCs, which are uniquely marked by the transcription factor ZBTB46. Development of cDC2s and cDC1s from the pre-cDC is dependent on IRF4 and BATF3/IRF8, respectively. Cell surface markers used for identification of each cell type are listed.

DCs are a heterogeneous population of cells, which can be classified as conventional DCs (cDCs) and non-conventional DCs (plasmacytoid DCs, monocyte-derived DCs, and Langerhans cells) (Figure 1). cDCs are the primary population responsible for naïve T cell activation and they express the transcription factor ZBTB46 (16, 17). cDCs can be further divided into two subsets based on ontogeny: type 1 cDC1s that develop in a BATF3/IRF8-dependent manner and type 2 cDC2s that are IRF4-dependent (18). These two cDC populations differ in cell surface marker expression, cytokine production, antigen processing and reside in distinct locations at steady state (18). Tissue cDC1s and cDC2s survey for infection or host damage, which if detected, induces DC migration to draining LNs. In contrast, LN-resident cDC1s and cDC2s acquire antigen that drains via lymphatics into LNs or is carried to LNs by migratory cells (19). These distinctions make each cDC subset specialized to drive particular T cell responses (20–22).

Non-conventional DCs are more diverse in their ontogeny and function. Plasmacytoid DCs (pDCs) are a unique subset that sense viral and bacterial pathogens and release high levels of type I interferons (IFN-I), stimulating both innate and adaptive immune cells. However, in comparison to cDCs, pDCs have a limited potential for antigen presentation (23). In mice there are two main monocyte subsets: inflammatory monocytes and patrolling monocytes (24). Inflammatory monocytes, including monocyte-derived DCs (moDCs), are recruited to infected tissue where they produce inflammatory cytokines to drive local and systemic inflammation. Patrolling monocytes reside in the vasculature where they regulate homeostasis of the endothelium and promote the resolution of inflammation in damaged tissues. Finally, Langerhans cells (LCs), which have little to no expression of ZBTB46, are considered part of the macrophage lineage and are self-renewing in the epidermis. In fitting with this distinction, LCs have important functions within the tissue where they modulate the properties of other immune cell types and contribute to tissue homeostasis. However, LCs also share functional overlap with cDCs, in that they are able to acquire antigen in peripheral tissues, migrate to the lymph nodes and activate naïve T cells (25).

## Which DC subsets control Tfh differentiation?

While DCs have been shown to prime the first stage of Tfh cell differentiation, much debate exists around the exact DC subset that is primarily responsible for driving this response *in vivo*. A likely major reason for this controversy is differences in the approaches used. These include the site of immunization, nature of the antigen used, the dose administered, timepoint of analyses, use of antigen targeting and the kinetics of the response. Given the right experimental conditions, most DC subsets can prime Tfh cells; however, the relative contribution of each subset to the generation of humoral responses under physiologic conditions remains less clear. Below we review the current literature describing the evidence for each DC subset in Tfh priming and provide a discussion of the interpretations and caveats for each in the context of the experimental systems used.

## Lymph node-resident vs. migratory DCs in Tfh priming

As described previously, DCs are found both in non-lymphoid tissues like the skin, lung and gut, as well in SLOs. Both migratory and LN-resident DCs have been implicated in driving humoral responses. Most studies comparing these DC subsets were performed using antigens administered in the skin, either intradermally, or sub-cutaneously (including footpad immunization). Following immunization, antigen-bearing migratory DCs arrive in the lymph nodes after 18–24 h (26). However, injection of antigen into the footpad or ear pinnae results in an almost instantaneous delivery of antigen to the draining LN, perhaps due to the pressure induced by injection into a limited tissue space (26–31), bypassing the need for antigen delivery by migratory DCs. It is important to bear this caveat in mind when interpreting experiments using these routes of immunization. Nevertheless, this route has been used to dissect the relative contribution of LN-resident versus migratory DCs to Tfh priming in a model referred to as the “van Gogh approach” (29). In this model, antigen is delivered intradermally in the ear pinnae of mice followed by immediate resection of the injection site. This effectively eliminates migratory DC-dependent antigen transport to lymph nodes, limiting humoral responses to those driven by LN-resident DCs. Resecting the ear actually does not impact the total amount of antigen reaching the lymph nodes because the majority is delivered immediately via lymphatics (31). Using this approach, different studies demonstrated strikingly different results.

Intradermal vaccination in the ear pinnae with UV-inactivated influenza resulted in similar antibody responses and protection against a lethal dose of influenza in van Gogh mice as compared to controls without resection. While Tfh responses were not evaluated, the study showed that a subset of LN-resident cDC1s and cDC2s acquire viral antigens within 40 min of immunization, migrate to the inter-follicular regions of the lymph nodes and engage with antigen-specific T cells (29). These results were reproduced in a similar study by Tozuka et al. who demonstrated that intradermally administered fluorescently-labeled antigen was acquired by a population of CD11b-expressing DCs (presumably the LN-resident cDC2s) within 30 min of immunization (28). In this study, they also showed that ear resection immediately after (<5 s) intradermal administration of influenza HA vaccine did not impact antibody responses to the vaccine (28). Gerner et al. used advanced microscopic techniques as well as the van Gogh approach to demonstrate that LN-resident cDC2s were important for Tfh cell priming (27). Although immunization with OVA-coated beads and CpG did generate significant antibody responses in van Gogh mice, the levels of the OVA-specific IgG were significantly higher in control mice, presumably augmented by the action of migratory DCs (27). Together these studies suggest that LN-resident cDC2s are sufficient for Tfh priming under experimental conditions which circumvent the requirement for antigen delivery through DC migration.

In contrast to these three studies, also using the van Gogh model, Levin and colleagues showed that migratory DCs are required to drive Tfh and B cell responses to HIV p24-coated nano-particles and that LN-resident DCs could not support either Tfh or GC B cell dependent antibody responses (31). Using antigen encapsulated in large beads that cannot free drain into lymph, we recently demonstrated that migratory cDC2s were sufficient to induce Tfh cell priming and antibody production (30). In this model, LN-resident DCs could cooperate with migratory DCs through antigen transfer, but they were not capable on their own to induce Tfh priming.

So why these discrepancies between the studies? One explanation could be the nature of the antigen used. The studies by Woodruff et al. (29) and Tozuka et al. (28) used influenza as a model system either employing UV-inactivated influenza or influenza-derived HA protein, respectively. Resident DCs might have a higher affinity for acquiring influenza antigen and thus use of these antigens might better target LN-resident DCs. Indeed, Gonzalez and colleagues demonstrate that a SIGNR1^+^ DC subset located in the medullary sinus of the LN preferentially bound UV-inactivated PR8 strain of influenza and migrated toward the B cell follicles (32). These DCs are most likely LN resident cDC2s that are located near the lymphatic sinus (27). However, it is important to note that blocking PR8 uptake by SIGNR1^+^ DCs did not significantly impact specific antibody responses to influenza (32).

It is also possible that not all antibody responses generated against influenza are Tfh cell-dependent. In line with this, anti-influenza IgG2b/c but not IgG1 antibodies are generated in Tfh cell-deficient mice and confer protective immunity to mice upon lethal influenza challenge (33). This study suggests that while IgG1 responses require a germinal center phase, Th1 cells are sufficient to provide B cell help for extrafollicular IgG2 induction against influenza (33). Indeed, in the study by Woodruff et al. they do observe a trend for decreased anti-influenza IgG1 but not IgG2b in van Gogh mice as compared to control mice (29).

**Figure 2:**
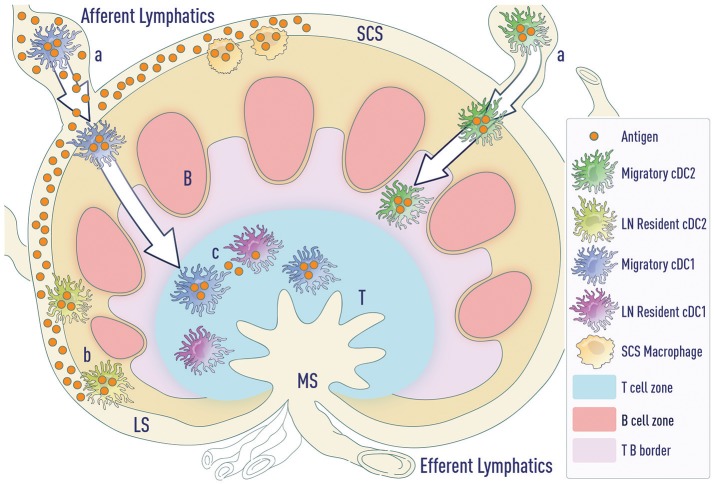
Modes of antigen access by DCs. **(a)** Migratory cDCs (and LCs—not depicted) phagocytose antigen in the tissue and then migrate to the LNs. cDC1s migrate to the T cell zone whereas cDC2s migrate to the T-B border. **(b)** LN-resident cDC2s lining the lymphatic sinus phagocytose free draining antigen from the lymphatics. **(c)** Antigen transported by migratory DCs can be transferred to resident DC subsets in the LNs. LS (lymphatic sinus), SCS (sub-capsular sinus), MS (Marginal sinus).

These data suggest that resident DCs can drive humoral responses under certain conditions, such as intradermal influenza vaccination (28, 29, 32). Under these conditions, a significant amount of antigen freely drains to the lymph node, bypassing the need for migratory cDCs. However, under limiting doses of antigen, LN-resident DCs may not be necessary for priming Tfh cells. A major hurdle in addressing this possibility is the lack of tools to selectively deplete LN-resident DC subsets while keeping migratory DCs intact. Further, distinct routes of antigen transport ensure that both resident cDCs and migratory cDCs access antigen and can present antigen to naïve T cells (Figure 2). This suggests that migratory cDCs and resident cDCs might regulate distinct steps of Tfh cell differentiation, as will be discussed later.

## Conventional DCs

cDCs have been shown to play a dominant role in priming T effector responses (23). Hence, it is not surprising that they also are implicated in priming Tfh cells. The recently identified transcription factor ZBTB46 is specifically expressed by cDCs and mice encoding a diphtheria toxin receptor under the *Zbtb46* promoter (*Zbtb46-DTR*) can be used to selectively deplete cDCs *in vivo* (16, 17). Using these mice, we and others have shown the loss of T-cell dependent humoral responses in the absence of cDCs (27, 34, 35). Both cDC1s and cDC2s have been shown to drive antibody responses (see below). Identification of the most relevant cDC subset for Tfh priming is complicated by the fact that there are migratory and LN-resident subsets of cDCs and—as described above–depending on the route of immunization, the organ system studied, and type of antigen used, both migratory and resident cDCs have been implicated in priming Tfh cells.

### cDC1s

Like LCs, skin-resident cDC1s, also express Langerin and hence some of the studies implicating LCs in humoral responses have also studied the role of cDC1s in mediating these responses. Using human *Langerin-DTA* mice (which lack LCs, but not cDC1s), Yao et al. demonstrated that targeting antigens to cDC1s (using antibodies against murine Langerin) in the skin is sufficient to prime Tfh cells. Further, cDC1s promoted humoral responses, albeit less efficiently than LCs in this model (36). Antigens can be efficiently targeted to cDC1s in the spleen via specific receptors such as CLEC9A or DEC-205. In two separate studies, Caminschi and colleagues demonstrate that targeting antigen (either OVA or Herpes Simplex Virus glycoprotein 1B) via CLEC9A, even in the absence of an adjuvant, primed efficient Tfh and GC B cell responses. Interestingly, they suggest that increased persistence of anti-CLEC9A mAb (and thus antigen) in the system drives enhanced CD4^+^ T cell activation and Tfh cell priming (37, 38). The authors noted that DEC-205 targeting is not as efficient in priming humoral responses especially in the absence of an adjuvant, potentially due to enhanced clearance of the mAb from circulation (38). These results were also reproduced in a separate study by Shin and colleagues (39).

In contrast, Levin and colleagues show that while depletion of LCs does partly abrogate Tfh cell and GC B cell responses to HIV p24 coated nano-particles, additional depletion of cDC1s has no further impact on these responses (31). In a similar approach, Kumamoto et al. using murine *Langerin-DTR* mice (to deplete both LCs and cDC1s) also show that cDC1s do not drive antibody responses to OVA and papain immunization in the skin (40). *Batf3*^−/−^ mice, which fail to develop cDC1s also have no defect in (and in some cases enhanced) Tfh and antibody responses to inhaled (30) and systemic antigens (34, 35). These results suggest that while targeting antigen to cDC1s could drive humoral responses, cDC1s are not necessary to prime Tfh and GC B cell responses to untargeted antigens, as in the case of vaccination and infection.

### cDC2s

It is well documented that irrespective of the organ system, cDC2s are superior to cDC1s in their ability to prime CD4^+^ T cells rather than CD8^+^ T cells (20, 34, 41, 42). Similarly, several reports show that cDC2s are the dominant Tfh-priming DC subset (27, 30, 35, 39), potentially due to their unique localization (30, 43). Tfh cell priming occurs in the T cell-B cell border (44), which includes the interfollicular zone (IFZ). We and others have clearly demonstrated that cDC2s occupy the T-B border regions in the lymph nodes (30, 43, 45) and the spleen (35, 46–49), suggesting that this subset of DCs is ideally positioned to prime Tfh responses.

A number of studies, including ours, have investigated the role of splenic cDC2s in driving humoral responses to blood-derived antigens. Using DC-specific IRF4 knockout mice, we demonstrated that splenic cDC2s, but not cDC1s drive allo-antibody responses to transfused red blood cells (RBCs) (34). Similarly, EBI2, a Gαi-coupled receptor, is required by cDC2s to position themselves in the bridging channels of the spleen, and *Ebi2*^−/−^ mice have impaired GC B cell responses to transfused sheep RBCs (48, 49). In line with this, the same authors in another report demonstrate that mice deficient in cDC2s (*Cd11c*^*Cre*^ *Irf4*^−/−^and *Cd47*^−/−^), but not cDC1s (*Batf3*^−/−^), also have impaired Tfh responses to sheep RBCs (35). In agreement, but using an alternative approach, Shin and colleagues showed that targeting antigens to cDC2s using antibodies against the cDC2-specific cell surface marker DCIR2 efficiently induced Tfh cell, GC B cell, and antibody responses to OVA (39, 47).

We recently reported that cDC2s also play a critical role in driving Tfh-dependent humoral responses (30). We showed that DC-specific deletion of the guanine nucleotide exchange factor *Dock8* results in impaired migration of cDC2s in the skin, lungs, and the spleen (30, 46, 50). DOCK8-deficient mice, but not *Batf3*^−/−^ (that lack all cDC1s), have defective Tfh cell responses to antigen administered sub-cutaneously, intravenously, or intranasally. As discussed previously, antigen targeting to dermal cDC1s has been shown to efficiently induce Tfh cell responses (36). However, the skin contains far fewer cDC1s than cDC2s and hence, antigen administered sub-cutaneously is not efficiently transported by cDC1s (30). In fact, most of the antigen administered sub-cutaneously is transported to the LN by migratory cDC2s. In order to compare migratory cDC1s to cDC2s, we administered antigen intranasally and found that both migratory cDC subsets efficiently transported antigens to the mediastinal LN. However, as in the skin, DC-specific DOCK8-deficient mice had impaired Tfh cell priming as well as impaired antigen-specific humoral responses to both OVA and influenza (as measured by antigen-specific IgG and weight loss in response to a lethal influenza infection). We noted similar defects in *Cd11c*^*Cre*^ *Irf4*^−/−^ mice which lack cDC2s. In contrast, loss of cDC1s in BATF3-deficient mice did not impact OVA-specific Tfh cell differentiation. In a recent study, Kumamoto et al. used *Mgl2-DTR* mice to ablate a subset of cDC2s which express CD301b. They determined that CD301b^+^ cDC2s inhibit Tfh cell responses to antigens administered with Th2-promoting adjuvants such as papain, but not in response to Th1 adjuvants like CpG (40). Although the mechanism remains to be determined, this work highlights that the major DC subsets outlined in Figure 1 are likely more heterogeneous than currently appreciated.

While these reports suggest that there is some consensus about the proficiency of cDC2s in Tfh cell priming, discrepancies do exist, especially regarding the role for migratory versus LN-resident cDC2s. In an elegant study using an advanced multi-parameter microscopic technique called histo-cytometry, Gerner and colleagues showed that migratory cDC2s occupy the IFZ (part of the T-B border) in inguinal LNs, while resident cDC2s are found in the lymphatic/medullary sinus regions (27). Based on this observation, one could hypothesize that migratory cDC2s are better positioned to drive Tfh responses; however, this study found that migratory cDC2s were not required for Tfh induction. Again, this was using methods of immunization that might bypass normal trafficking routes such as intra-auricular injection (31). To distinguish the migratory cDC2s from resident cDC2s (and cDC1s), we used OVA-encapsulated beads that cannot free drain to the lymph nodes via the lymphatics and hence can only be transported by migratory cDCs. Administration of these beads to DOCK8-deficient mice resulted in impaired Tfh cell responses, re-emphasizing the inability of cDC1s to drive these responses. In BATF3-deficient mice, cDC2s are unaffected and administration of beads to these mice restricts antigen to migratory cDC2s. Tfh cell frequencies in BATF3-deficient mice were normal as compared to WT controls, indicating that migratory cDC2s are sufficient to prime Tfh cell responses (30).

The overall conclusion from these studies is that cDC2s play a dominant role in priming Tfh cells. However, as will be discussed next, other non-conventional DCs likely partner with cDC2s to promote early phases of T cell activation. The discrepancies stated above, however, suggest a greater level of heterogeneity than currently appreciated among cDC2s. Future studies to selectively target different subsets of cDC2s including migratory, resident and CD301b-expressing cells will help clarify positive and negative influences on Tfh differentiation.

## Non-conventional DCs

### Monocyte-derived DCs

Using the van Gogh model Levin et al. show that while ear resection does not impair the frequency of monocyte populations in the draining lymph node, it does result in impaired Tfh responses to HIV p24 coated nano-particles (31). Similarly, Kumamoto et al. showed that depletion of monocyte-derived DCs using anti-Gr1 antibodies also had no impact on Tfh responses (40). Together, these results suggest that monocyte-derived DCs are not required for priming Tfh cells.

However, using different models, other groups have come to the opposite conclusion. Barbet et al. show that Tfh responses to intraperitoneal *E. coli* vaccination are TRIF-dependent, and driven by CD11c^+^ CX3CR1^+^ “patrolling” monocytes. They demonstrate that TRIF-dependent Tfh priming is unaffected in the absence of cDCs (using *Zbtb46-DTR* mice) or inflammatory monocytes (*Ccr2*^−/−^ mice) (51). Immunization with a combination of CpG-B and incomplete Freund's adjuvant (IFA) results in higher frequencies of Tfh cells as compared to IFA alone (52). Using *Ccr2*^−/−^or *Cx3Cr1*^−/−^mice, Chakarov et al. generated mice with deficiencies in moDCs but not cDCs to demonstrate that the enhanced Tfh response induced by CpG-B is driven by IL-6-producing moDCs (52). However, given that a significant frequency of Tfh cells were induced in the absence of moDCs (52), one conclusion could be that, while cDCs play a dominant role in priming Tfh responses, moDCs enhance this response via IL-6 production. Interestingly, Germain and colleagues demonstrated that administration of high doses of DT to *Zbtb46-DTR* mice triggers infiltration of CD11b-expressing monocytic populations that are distributed throughout the lymph node (27). Thus, while these cells may not form part of the canonical Tfh differentiation pathway, these results together indicate that under certain conditions, moDCs can occupy similar functional niches as cDC1s and cDC2s and can help prime Tfh responses.

### Langerhans cells

The absolute requirement of LCs for humoral responses has been addressed by several studies using Langerin-diphtheria toxin receptor mice (31, 40, 53). To delineate which migratory DC subsets play a role in Tfh responses, Levin et al. used *Langerin-DTR* mice to deplete Langerin-expressing DCs i.e., LCs and cDC1s in the skin (31). Post DT treatment, LCs remain depleted for more than 2 weeks while cDC1s are replenished within 1 week. They employed this differential response to generate mice lacking only LCs (DT administered 2 weeks prior to immunization) or both LCs and cDC1s (DT administered 2 days prior to immunization). Loss of either LCs alone or both LCs and cDC1s decreased but did not abrogate Tfh and antibody responses to HIV p24-coated nano-particles that were administered in the absence of any other adjuvant. The authors conclude that while LCs do play a role in Tfh-dependent B cell responses, migratory cDC2s also contribute to humoral responses in the skin (31). Corroborating these results, in a model using OVA and the adjuvant papain, Kumamoto et al. demonstrate that LCs are not required for humoral responses to cutaneous antigens (40).

In an earlier study using the same *Langerin-DTR* mice, Zimara et al. demonstrated that loss of LCs results in impaired Tfh induction and early antibody responses (day 10) to *Leishmania major* infection (53). However, overall *Leishmania*-specific humoral responses (i.e., day 40 post-infection) remain unaffected. The study further showed that the size of GC is decreased in mice lacking LCs. These impaired responses are, however, restricted to *Leishmania* infection and not seen with other T-dependent antigens like DNP-KLH (with the adjuvant aluminum hydroxide). The authors suggest that *Leishmania* infection, unlike DNP-KLH and alum, does not lead to maturation of cDCs and under these circumstances, LCs drive humoral responses (53).

Yao et al. used transgenic mice expressing human Langerin (*huLangerin*), specifically in LCs but not in other DCs (i.e., dermal cDC1s, where murine Langerin is expressed) (36). Using monoclonal antibodies specific to human Langerin, the authors demonstrate that targeting antigens to LCs *in vivo* efficiently induces antigen-specific Tfh cell responses. The response generated was dose-dependent and was only generated against foreign antigens (and not self-antigens like MOG peptide). Targeting LCs either in the skin and LNs (systemic administration) or in the skin alone (topical application) efficiently induced B cell responses including GC B cell formation and a protective humoral response against lethal influenza challenge (36). It is interesting to note that as compared to previously published reports (7, 44), the kinetics of the response generated by targeting LCs is slightly delayed, i.e., the peak of the Tfh cell response observed is around day 7 and the peak of GC B cell expansion is around day 14 (36). One potential explanation for this is that LCs are known to have significantly slower migration kinetics with their numbers peaking 3–4 days post-immunization (even under inflammatory conditions) as opposed to cDCs that reach the draining cutaneous lymph nodes 18–24 h post-immunization (50, 54, 55).

In the work by Yao et al. LCs were shown to primarily drive humoral responses when antibodies to Langerin were administered without an adjuvant (36). Under these circumstances, the lack of adjuvant would fail to efficiently induce maturation and migration of migratory cDCs- a pre-requisite for their ability to induce effective T cell responses. Indeed, even under inflammatory conditions, expression of co-stimulatory molecules remain unchanged on LCs that migrate to the LN, suggesting that under steady state, LCs have a mature phenotype (54). In contrast, migratory cDCs have higher expression of co-stimulatory molecules and emigrate in greater frequencies upon maturation (30, 56) possibly indicating that these cells play a dominant role under inflammatory conditions such as in the case of an infection or vaccine response. These results together suggest that LCs can drive humoral responses to low-abundance, weakly immunogenic antigens that do not efficiently induce cDC maturation (36, 53).

## DC-dependent factors regulating early Tfh differentiation

Early differentiation signals required for Tfh cells have been extensively characterized. Signals that function early in the Tfh differentiation process, and that are independent of B cells, have frequently been ascribed to DCs. However, it is important to note that there is still limited evidence that directly proves that these are DC-unique factors. This is further complicated by the fact that multiple DC subsets could play a role in the differentiation process.

### DC maturation and pattern recognition receptors

DCs must undergo a maturation process for the induction of a productive adaptive immune response. Almost 30 years following Charles Janeway's proposal, several PAMPs (pathogen-associated molecular patterns) or DAMPs (danger-associated molecular patterns) have been identified that are detected by the immune system via specific groups of germ-line encoded receptors called Pattern Recognition Receptors (PRRs). These include Toll-like receptors (TLRs), C-type lectin receptors (CLRs), NOD-like receptors (NLRs), RIG-I-like receptors (RLRs) and AIM2-like receptors (ALRs). Engaging these receptors results in DC maturation and thereby an effector T cell response (57).

TLR agonists have widely been used in induction of Tfh responses. Notably, TLR3 (39, 58), TLR4 (5, 30, 39, 58) and TLR9 (27, 40, 52) agonists have been shown to effectively drive Tfh responses in both mice and humans. Kumamoto and colleagues show that the immunosuppressive effects of CD301b^+^ cDC2s is only seen using “Th2-type” adjuvants like papain and that engagement of TLRs like TLR4 and TLR9 overcomes the Tfh-inhibiting capacity of this DC subset (40). In a recent study, Ugolini et al. demonstrate that TLR8 on human monocytes senses microbial viability by binding to bacterial mRNA. The activation of monocytes by this special class of PAMPs called “vita-PAMPs,” results in production of IL-12 which in turn drives BCL6 expression and IL-21 production by CD4 T cells (59). Barbet et al. reported that in mice, bacterial viability is detected by CX3CR1^+^ monocytes via a TRIF-dependent mechanism. The downstream Type I IFN response along with inflammasome activation drives Tfh differentiation (51). While the role of IFN signaling in driving Tfh responses is discussed later, it is important to note that TLR3 and TLR4 agonists also drive Tfh responses by inducing an autocrine Type I IFN signal in DCs (58). Using human monocyte-derived DCs, Schmitt et al. compared Tfh inducing capacities of different TLR agonists and show that TLR4, TLR5, and TLR7/8, but not TLR2, activation induces IL-21 production from CD4^+^ T cells, with TLR4 being the most potent followed by TLR5 and TLR7/8 (5).

Monoclonal antibodies against C-type lectin receptors (CLRs) have been used to target antigens to specific DC subsets *in vivo* and, as described previously, several studies have employed this approach to study the Tfh-priming capacities of different DC subsets. Targeting antigen to certain C-type lectin receptors is alone sufficient to prime Tfh cells and does not require additional adjuvants. For example, targeting LCs via the CLR Langerin does not result in LC activation but is sufficient to drive Tfh cell differentiation *in vivo* (36). Similarly, Caminschi and colleagues demonstrate that targeting antigen to cDC1s via the CLR CLEC9A induces a robust Tfh cell response (37, 38) and this response is not augmented by co-administering the TLR9 agonist, CpG (38). In contrast, Shin et al. report that effective Tfh cell differentiation is observed when antigen is targeted to cDC2s via the CLR DCIR2 in the presence of TLR3 (Poly I:C) or TLR4 (LPS) agonists (39).

There are a limited number of studies addressing the role of other groups of PRRs in driving Tfh differentiation. While the role of NLRs in priming Tfh cells has not been directly addressed, alum, a potent activator of the NLRP3 inflammasome (60, 61), has been used as an adjuvant in several studies (6, 62, 63). Further, IL-1β, an effector cytokine produced downstream of inflammasome activation (57), also plays a role in priming Tfh cells, as will be discussed later. Regarding the role of RLRs, one study demonstrated that co-administering influenza antigens with 5′ ppp-double-stranded RNA, a RIG-I ligand, enhances Tfh differentiation and antibody responses to influenza via a Type I IFN-dependent mechanism (64).

### Antigen presentation

The strength and duration of antigen presentation plays a critical role in determining the outcome of CD4^+^ T cell responses, i.e., T effector cells versus Tfh differentiation (65). Using a pigeon cytochrome C (PCC) model, Fazilleau et al. demonstrated that T cells with higher antigen affinity preferentially differentiate into Tfh cells (66). Tfh cells have stronger affinity for peptide-MHC II complexes and a more restricted TCR repertoire as compared to T effector cells (66). In addition to TCR affinity, increased TCR signals (using high antigen concentrations) are required for maximal IL-21 production (66). Moreover, increasing the antigen dose (11) or gradually increasing antigen administration over 2 weeks (67) boosts the generation of both Tfh and GC B cells. Thus, antigen dose is tightly linked to both Tfh induction and the magnitude of GC responses. In contrast, both high and low affinity antigen-specific T cell clones equally differentiate into Tfh cells in mice immunized with Friend's virus (68). This study used a chronic retroviral infection model and the authors suggest that the differences in their study as compared to Fazilleau et al. is probably due to antigen availability. Thus, they hypothesize that under conditions of limiting antigen availability, high TCR avidity would drive Tfh cell differentiation (68). However, TCR avidity, as in the case of the previous study, does impact IL-21 production, suggesting that some features of Tfh differentiation are indeed TCR-intrinsic (68).

Nevertheless, the contrasting results of these studies suggest that simple affinity of TCR to peptide-MHC II complexes, i.e., the receptor occupancy model, would not explain Tfh versus T effector cell differentiation. An alternative kinetic proofreading model suggests that the duration of interaction between TCR and peptide MHC II complexes (i.e., DC-T cell interaction times) is a better predictor of the outcome of the T cell response. Using single cell clones, Tubo and colleagues elegantly provide support for this model. They show that longer dwell time between TCR and peptide-MHCII, rather than TCR affinity, preferentially drives Tfh cell differentiation (69). In a recent study, Benson et al. visualized this process *in vivo* to show that the time of antigen presentation by DC to CD4^+^ T cells is critical for Tfh differentiation *in vivo* (7). Immunizing mice sub-cutaneously with 200 nm sized antigen-coated nanoparticles efficiently primed Tfh responses and protective antibody responses to influenza. Using multiphoton imaging, DC and antigen-specific CD4^+^ T cell interactions were imaged *in vivo*. The authors defined 3 stages of DC-T cell interaction over the course of the immune responses: Stage 1 (0–8 h), Stage 2 (12–24 h), and Stage 3 (48–72 h) post-immunization. Interactions longer than 10 min between DC-CD4^+^ T cells at Stage 3 were required for efficient Tfh cell priming. Disrupting MHCII-TCR binding at this stage impaired Tfh cell frequencies, suggesting that sustained antigen presentation is required for Tfh cell differentiation (7). These results together indicate that DC subsets that stably express antigen-MHCII complexes are probably superior at priming Tfh cells. Since cDC2s are more efficient than cDC1s in processing antigen for MHCII, this could explain why this subset seems to be more effective in Tfh priming, as discussed above (20, 70). However, experimental models in which antigen is specifically targeted to cDC1s could compensate for this difference and thereby enhance the ability of cDC1s to promote Tfh differentiation (37, 38).

### Co-stimulatory molecules

Tfh priming requires a variety of co-stimulatory molecules including B7 family members, CD40L, OX40L, and ICOSL (63). The most extensively studied co-stimulatory molecule with regards to Tfh priming is OX40L. However, studies have revealed, at least in part, that CD28 engagement and/or CD40L engagement leads to upregulation of OX40 on T cells (63).

Early reports demonstrated that CD28-deficient mice have impaired GC and humoral responses (71). Mice overexpressing CTLA4 (mCTLA4-Hγ1 transgene), the inhibitory ligand for CD28, also showed similar impairment in T-dependent B cell responses (72). However, Lane and colleagues in later studies demonstrated that the loss of these humoral responses was primarily due to impaired OX40 expression. OX40 is upregulated on naïve T cells following CD28 activation and activation of T cells by OX40L promotes expression of IL-4 and CXCR5 (73, 74). Interestingly, constitutive expression of OX40L by DCs (using CD11c-OX40L transgenic mice) leads to increased Tfh cell differentiation; however, this is also dependent on CD28 signaling (74). A recent study by Watanabe et al. using Cre-mediated deletion of CD80 and CD86 in DCs illustrated that CD28 signaling at the stage of T-DC interaction is critical for initial priming and expansion of T cells (75). In contrast, loss of CD80 and CD86 in the B cell compartment did not affect the generation of Tfh cell or GC B cells nor humoral responses in terms of affinity maturation and serum IgG levels (75). Collectively, these studies demonstrate a critical role of DCs in delivering CD80/CD86 co-stimulatory molecules during pre-Tfh differentiation for optimal Tfh and GC responses.

Patients with CD40L deficiency as well as CD40-deficient mice show impaired Tfh cell frequencies (76, 77). Given that CD40 activation leads to OX40L expression on both murine and human DCs (78, 79), the Tfh defects seen in the absence of CD40 could indeed be a downstream effect of abrogated OX40/OX40L signals. In line with this, Fillatreau et al. report that *Cd40*^−/−^ mice, like *Ox40*^−/−^mice, have impaired accumulation of T cells in follicles. CD40 is required for CXCR5 expression in T cells (80). Restoring CD40 expression in DCs but not B cells, either using mixed bone marrow chimeras or by adoptive transfer of CD40^+^ DCs, restores this response in *Cd40*^−/−^ mice. Furthermore, treating *Cd40*^−/−^ mice with OX40L-huIgG1 fusion proteins readily rescues CD4^+^ T cell migration into follicles (80). However, administration of agonistic OX40 antibodies during LCMV infection diverts Tfh differentiation to T effector differentiation by inducing Blimp-1 expression (81), suggesting that the role of OX40 signaling in Tfh differentiation is context-dependent.

These results suggest that Tfh priming is primarily regulated by OX40 signaling downstream of CD28 and CD40L. However, Akiba et al. compared the effects of all three co-stimulatory molecules on Tfh priming *in vivo* and clearly demonstrate that while CD28 and CD40 are indeed required for Tfh cell differentiation and GC B cell responses, the requirement for OX40 signaling is strain- and immunization site-specific (76). The authors immunized various mouse strains, including BALB/c and C57BL/6, at different sites and compared OX40 expression on Tfh cells in the spleens and in skin-draining LNs. They show that OX40 blocking antibodies only impair GC and Tfh responses in LNs of C57BL/6 mice but not in BALB/c mice, possibly since OX40L expression is observed only in LN but not splenic Tfh cells in C57BL/6 mice post-immunization (76). The requirement for CD28, beyond OX40 upregulation, in early Tfh cell responses is further demonstrated in a study by Smith and colleagues (82). *Cd28*^*flox*/*flox*^ *Ox40*^*Cre*/+^ mice were used to block CD28 signaling after T cell priming and expansion. The authors show that these mice have reduced frequencies of Tfh and GC B cells in response to intranasally administered influenza A, as compared to control mice. Loss of CD28 in activated T cells, results in increased apoptosis and impaired BCL6 and ICOS expression in Tfh cells (82). These results suggest that persistent CD28 stimulation, beyond early naïve T cell activation, is required for Tfh cell differentiation and maintenance as well as functional humoral responses (82).

A recent study by Tahiliani et al. demonstrates that in a murine model of vaccinia virus infection, OX40-deficient mice have impaired Tfh and B cell responses (83). Blocking OX40L during and after Tfh cell generation leads to a significant reduction in Tfh, GC Tfh and GC B cell frequencies (83). Finally, OX40L-expressing DCs were seen in bridging channels in the spleen and also co-localized with OX40^+^ T cells, suggesting that cDC2s could in part provide some of these signals during Tfh cell priming (83). Further, TSLP-activated human DCs prime IL-21- and CXCL13-producing CXCR5^+^ PD-1^+^ Tfh cells *in vitro*. Blocking OX40L, but not ICOSL, in this system reduces IL-21 and CXCL13 production. It is important to note that while BCL6 expression is reduced in CXCR5^hi^ PD1^hi^ cells, blocking OX40L in this system does not reduce Tfh cell frequencies (15). Together, these results indicate that the requirement for OX40L during Tfh cell differentiation is not absolute, but under specific conditions, could regulate certain facets of Tfh cell differentiation.

ICOS signaling plays a central role in Tfh cell priming. ICOS-deficient mice and humans have impaired Tfh cell and GC B cell frequencies, reduced T cell localization to follicles and impaired humoral responses (76, 77). Roquin 1 and Roquin 2 are RNA-binding proteins that play an important role in post-transcriptional repression of ICOS expression. Combined loss of Roquin 1 and Roquin 2 (84, 85), or loss of mir-146a (also a negative regulator of ICOS) (86) specifically in T cells, increases ICOS expression, resulting in spontaneous accumulation of Tfh and GC B cells in mice (84–86). B cell-specific ablation of ICOSL results in a similar loss of Tfh cell differentiation as ICOSL knockout mice, which suggests that ICOS signaling primarily plays a role during the B cell phase of Tfh priming (87). Similarly, blocking ICOSL *in vitro* did not impair human Tfh cell differentiation by TSLP-activated DCs (15). In contrast, Choi et al. demonstrate that early ICOS signaling is required for BCL6 expression and Tfh cell commitment as *Icos*^−/−^ T cells failed to differentiate into Tfh cells as early as day 3 post-immunization (88). The authors also show that loss of B cells had no impact on Tfh cell frequencies 3 days post-immunization, suggesting that this stage was B cell-independent. Further, adoptive transfer of antigen-loaded DCs was sufficient to induce BCL6 and CXCR5 expression in T cells confirming that this early stage of Tfh cell differentiation is indeed DC-dependent. The impaired differentiation of *Icos*^−/−^ T cells to Tfh cells early in the response suggests that ICOSL signaling by DCs is critical for Tfh cell priming. Further, ICOS signaling during this first stage is required for BCL6 expression by Tfh cells, which the authors show in turn is critical for CXCR5 expression (88). Together, these findings highlight that ICOS is important for multiple stages of Tfh differentiation and is provided by both DCs and B cells.

Finally, NOTCH signaling also regulates Tfh cell differentiation. Loss of NOTCH 1 and 2 specifically in CD4^+^ T cells leads to reduced Tfh cell frequencies, impaired IL-4 production by Tfh cells and concomitantly, reduced GC B cell and IgE responses (89, 90). While NOTCH signaling controls IL-4 production by Tfh cells (90), NOTCH-deficient Tfh cells also fail to downregulate BLIMP1 (PRDM1) or upregulate BCL6, Cmaf, and IL-21, in an IL-4-independent manner (89). DC-specific deletion of the NOTCH ligand, the E3 ubiquitin ligase Mind bomb1 (MIB1) also impairs early Tfh cell differentiation. Tfh cell frequencies in these mice are eventually comparable to controls at later stages of the response indicating that requirement for DC-derived NOTCH signals is not absolute. The authors also demonstrate that depletion of NOTCH ligands on B cells and follicular DCs has no impact on Tfh cell priming (90).

### Cytokines

The cytokine milieu in SLOs plays a critical role in polarizing T cells either toward Tfh or other T effector fates. Depending on the PAMPs or DAMPs associated with the antigen encountered, DCs secrete cytokines that could significantly influence the outcome of the T cell response. Given that different DC subsets preferentially express distinct cytokines, this may specialize them for driving the polarization of different T cell subsets. Further, as discussed previously, distinct DC subsets occupy unique niches within the SLOs; therefore, a combination of DC-intrinsic differences in cytokine production in combination with cells in the niche could favor Tfh differentiation versus other T effector fates.

### IL-6

In the mouse, IL-6 is one of the first cytokine signals to influence the early stages of Tfh differentiation. IL-6 signals via STAT3 and this pathway induces early expression of BCL6 in T cells (91, 92). Deficiency of IL-6 or STAT3 impairs Tfh differentiation and antibody responses *in vivo* (91, 93). DCs produce copious amounts of IL-6 in response to stimulation with various PAMPs or CD40 activation (2). Adoptive transfer of antigen-pulsed IL-6-sufficient DCs but not IL-6-deficient DCs leads to efficient antibody responses *in vivo* (93). DC-specific deletion of *Blimp1* in mice results in a spontaneous lupus-like phenotype characterized by increased Tfh cell frequencies and autoantibody production (94). The authors demonstrate that BLIMP1 deficiency causes increased IL-6 production by DCs. Further, *Il-6* heterozygous DC-specific *Blimp1*-deficient mice (*Il-6*^+/−^ *DCBlimp1*^*ko*^), in which IL-6 expression is no longer elevated, have reduced GC and Tfh cell responses compared to *DCBlimp1*^*ko*^, suggesting that DC-derived IL-6 drives the responses observed in these mice (94). In a subsequent study, Kim et al. defined IL-6-dependent and -independent pathways by which BLIMP1 also regulates the expression of Cathepsin S, an endolysosomal protease which influences antigen processing, suggesting this as an additional mechanism for the observed lupus-like phenotype (95). They showed that DCs from BLIMP1-deficient mice induced IL-21 expression in co-cultured T cells and that this is abrogated in the presence of a Cathepsin S inhibitor (95).

These results indicate that DCs could indeed be the source of IL-6 for Tfh cell differentiation. However, other studies suggest that, rather than driving early Tfh cell differentiation, DC-derived IL-6 instead fine-tunes the phenotype of newly primed Tfh cells, with other cell types producing the IL-6 that is required for early Tfh differentiation. Using adoptively transferred IL-6-deficient antigen-pulsed DCs, Andris and colleagues show that DC-derived IL-6 has no impact on Tfh cell frequencies or CXCR5, PD1 and BCL6 expression (96). This is in line with a report from Chen et al. who, using mixed bone marrow chimeras, report that IL-6 deficiency in radio-resistant (such as stromal cells) but not radio-sensitive hematopoietic cells (such as DCs), impacts CXCR5 and BCL6 expression in Tfh cells (62). However, Andris and colleagues also show that IL-6-mediated STAT3 signaling suppresses GATA3 expression in Tfhs. Loss of DC-derived IL-6 results in decreased IL-21 and increased IL-4 production by Tfh cells, resulting in increased IgE responses (96). These results indicate that IL-6 production from stromal cells as well as DCs have non-redundant functions during Tfh cell differentiation.

### IL-12

IL-12 has been shown to play a critical role in Tfh cell differentiation in human T cells (5). Triggering of certain pattern recognition receptors like TLRs induces IL-12 production by DCs (2). In two separate studies, IL-12, and to a lesser extent IL-23, were able to induce IL-21 from human CD4^+^ T cells activated *in vitro* (5, 97). IL-21-expressing T cells express CXCR5 and ICOS and promoted B cell help *in vitro* (5, 97). Similarly, allogenic stimulation of T cells with DCs exposed to different heat-killed bacteria, resulted in IL-21 expression by T cells which in turn regulated antibody production by B cells *in vitro*. This process is IL-12-dependent, as inhibiting IL-12 *in vitro* abrogated these responses (5). Human cDC2s produce higher levels of IL-12 and IL-6 as compared to cDC1s in response to a variety of TLR ligands (98, 99). This might explain, in part, why TSLP-activated human cDC2s are superior to cDC1s in activating and inducing IL-21 production from T cells (15). Using adoptive transfer of DCs, Andris and colleagues demonstrated that loss of IL-12 does not impact murine Tfh cell differentiation (96). In contrast to human cDC2s, murine cDC2s actually produce less IL-12 (30, 100). Therefore, the role of IL-12 appears to be different in human vs. murine Tfh-DC interactions and further work remains to be done on what accounts for these species-specific effects.

### Type I IFN

Type I IFN and IL-27 modulate Tfh cell differentiation. Interferon-alpha/beta receptor-deficient (*Ifnar*^−/−^) mice have impaired Tfh cell and antibody responses as compared to control mice when immunized with NP-OVA and LPS (58). IFNAR deficiency in either DCs or radio-resistant cells also results in reduced frequencies of Tfh cells. Further, *Ifnar-*deficiency in DCs results in reduced IL-6 production (58). As discussed previously, IL-6 production from stromal cells and DCs seems to non-redundantly impact Tfh cell differentiation and these data further support this hypothesis.

While IFN-induced IL-6 production provides one potential mechanism, other pathways downstream of IFNAR signaling have also been shown to drive Tfh cell responses. Gringhuis et al. demonstrate that autocrine IFNAR signals in human DCs can promote IL-27 production that in turn drives Tfh cell differentiation (101). They show that treating human DCs with fucose, an agonist of the C-type lectin receptor DC-SIGN, activates IKKε, a member of the non-canonical IKK kinase family. This pathway synergizes with an autocrine Type I IFN signal to drive IL-27 production. The IL-27 produced by the DCs enhances BCL6 and IL-21 expression in T cells (101). In line with this, using *Il27r*α^−/−^ mice, Batten et al. demonstrated that IL-27 promoted IL-21 production and Tfh cell survival (102). A recent study by Blander and colleagues also demonstrated an alternative pathway by which Type I IFN regulated Tfh differentiation (51). They demonstrated that autocrine IFNAR signals promoted Caspase 1- and Caspase 11-mediated production of IL-1β by DCs. In T cells, IL-1β signals via IL1R1 and drives expression of BCL6, CXCR5, and ICOS. In addition, IFNAR signaling in Tfhs directly induces production of IL-21 (51). However, this study also shows that CX3CR1^+^ CCR2^−^ monocyte-derived DCs and not cDCs drive these Tfh responses (51). The presence of multiple pathways by which IFNAR signaling regulates Tfh cell differentiation raises the possibility that depending on the antigen encountered, different DC subsets could utilize alternative IFNAR-dependent pathways to prime T-dependent B cell responses.

### IL-2

IL-2 is a negative regulator of Tfh cell differentiation. IL-2 induces BLIMP-1 expression in T cells which suppresses BCL6 and downregulates CXCR5 (1). Further, IL-2-mediated mTORC1 activation of AKT also suppresses Tfh cell differentiation (103). Under certain stimuli, DCs have been shown to produce IL-2, a process that is counter-productive for Tfh cell priming (2). However, a recent study by Cyster and colleagues demonstrates that certain DC subsets like cDC2s express the IL-2 receptor alpha chain, CD25 (35). However, cDC2s do not respond to IL-2, as seen by STAT5 phosphorylation. The authors suggest that both soluble and secreted CD25 expression by cDC2s creates a “cytokine-sink” for IL-2. Limiting concentrations of IL-2 in the T-B border makes this niche then favorable for Tfh cell differentiation (35). However, in our studies, while we did find higher expression of CD25 on lung cDC2s, we could not find evidence supporting the role of DC-dependent CD25 expression on Tfh responses (30). Given that other cells in the T-B border including activated B cells express CD25 (104), we hypothesize that multiple cell types could cooperate to create an effective IL-2 sink.

## The three-step differentiation model

Spatio-temporal distribution of lymphocyte subsets within the SLOs are critical for efficient Tfh cell priming. Based on the literature reviewed here, the following conclusions can be drawn regarding DC subsets and Tfh priming.

**Figure 3:**
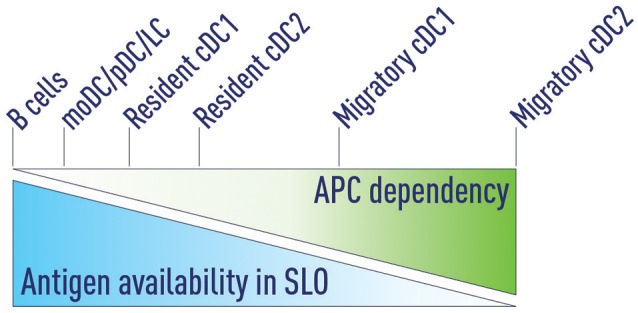
Antigen availability and APC dependency. We propose that antigen availability in secondary lymphoid organs (SLOs) determines which antigen presenting cells (APCs) are able to support Tfh priming. At very high antigen concentrations B cells can serve as the sole APC to support Tfh development. Decreasing antigen concentrations progressively increases the dependency on APCs that are more effective in priming Tfh cells. Migratory cDC2s are the most potent Tfh-priming APC and are both necessary and sufficient at low antigen concentrations.

1. DC subsets occupy distinct regions within lymphoid organs. Migratory cDC1s, resident cDC1s and LCs are found within the T cell zones, migratory cDC2s are located in the T-B border region (including the IFZ) and resident cDC2s reside in the lymphatic sinuses (30, 45).
2. Excluding antigens from certain DC subsets or depletion of specific DC subsets does not impair naïve T cell activation (27, 30, 31), suggesting that a redundancy exists in this step of T cell priming.
3. Antigen availability in SLOs determines which antigen presenting cells (APCs) are able to support Tfh priming. Decreasing antigen concentration progressively increases the dependency on APCs that are more effective in priming Tfh cells (Figure 3).
4. Distinct DC subsets can sequentially prime T cell responses (26).
5. Early after activation, naïve CD4^+^ T cells differentiate into BCL6^+^ pre-Tfh cells (9) and migrate to the T-B border - a process regulated by multiple factors including CCR7 (105), CXCR5 (44, 62, 105), and EBI2 (35). Pre-Tfh cells differentiate in the T-B region to Tfh cells (44). This process is initially B cell-independent and DC-dependent (6, 44, 63, 88).
6. Tfh cells interact with antigen-bearing activated B cells and mature into effector Tfh cells (1).

**Figure 4:**
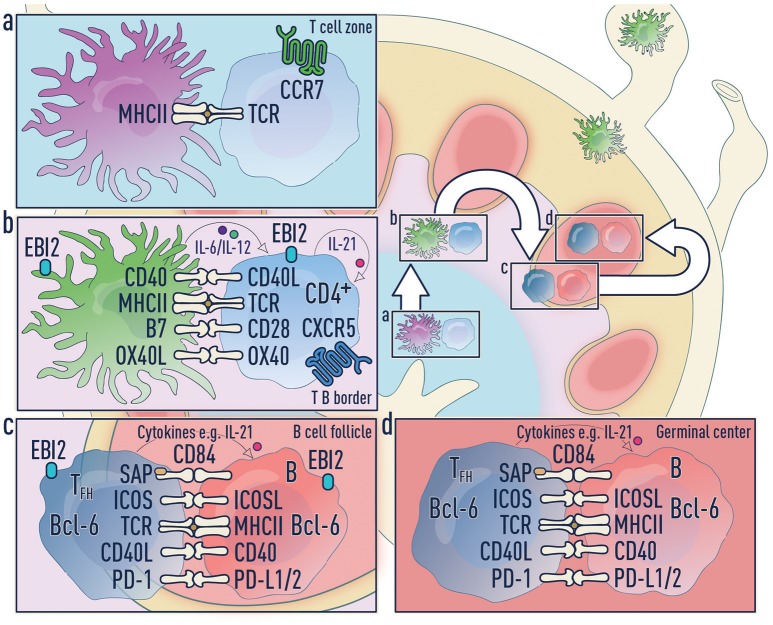
Three-step model of Tfh differentiation. **(a)** Step 1—Naïve T cell activation phase: Upon antigen acquisition any of the DC subsets or Langerhans cells can activate naïve T cells. These T cells downregulate CCR7 and upregulate CXCR5 allowing migration to the T-B border in an EBI2-dependent manner. It is unclear which co-stimulatory molecules and cytokines are required during this phase. **(b)** Step 2—Pre-Tfh phase: Migratory cDC2s home to the T-B border and this process is regulated by a number of factors including CCR7, CXCR5 and EBI2. Migratory DCs can accomplish both step one and step two. **(c)** Step 3—Tfh commitment phase: B cells are the major antigen presenting cell in this final step and provide signals to complete Tfh cell differentiation. **(d)** Tfh effector phase: Mature Tfh cells enter the germinal center where they promote survival, affinity maturation and class switch recombination of B cells.

Based on these data, we propose a three-step model for Tfh cell differentiation under conditions that deliver sufficient free draining antigen to LNs (e.g., high antigen doses, footpad or intra-auricular immunization, or infection within the LN) (Figure 4).

1. **Antigen transport and naïve T cell activation:** DCs can access antigens in three ways (Figure 2):
  - Migratory cDCs (and LCs) phagocytose antigen *in situ* and then migrate to the LNs
  - LN-resident cDC2s lining the lymphatic sinus endothelium phagocytose free draining antigen from the lymphatics
  - Antigen transported by migratory DCs can be transferred to resident DC subsets in the LNs
  - Any of these DC subsets upon antigen acquisition can activate naïve T cells. These T cells migrate to the T-B border in a CXCR5- and/or EBI2-dependent process.
2. **Pre-Tfh differentiation by migratory cDC2s:** Migratory cDC2s home to the T-B border and this process is regulated by a number of factors including CCR7 (106), CXCR5 (2, 30, 43) and EBI2 (48). As migratory cDC2s uniquely position themselves in the IFZ, they will efficiently support pre-Tfh differentiation. This makes cDC2s increasingly important when the level of antigen in the SLO is limiting. In the absence of free draining antigen, migratory DCs can accomplish both step one and step two.
3. **Tfh commitment by B cells:** As has been extensively described, B cells become the major antigen presenting cell in the final stage and provide signals including ICOSL/ICOS, CD40/CD40L, and CD84/CD84-SAP to complete Tfh cell differentiation (1, 2, 63).

## Summary and future directions

DCs are heterogeneous, and multiple subsets have been implicated in priming Tfh cells. Although our three-step model incorporates these findings, it is important to bear in mind that there is currently no definitive evidence clarifying the individual role of each of these DC subsets in Tfh cell differentiation. It is likely that the type and body location of immune insult determines the DC subsets responsible. The biggest hurdle toward addressing this is the ability to specifically deplete either resident or migratory cDC subsets *in vivo*. Identifying factors that uniquely drive the development, maturation, or migration of either resident or migratory cDC1s and cDC2s would be critical for the generation of such murine models.

While many Tfh cell differentiation factors including cytokines and co-stimulatory molecules have been described, it still remains unclear whether DCs are indeed the primary source of these factors. If so, which DC subsets provide them, at what stage during Tfh differentiation are these produced, where within the SLO are they secreted, and is this in conjunction with antigen presentation? Depletion of these factors within specific DC subsets would be one approach toward addressing these questions. Further, given that stromal cells and DCs appear to secrete some of the same factors that drive Tfh cell differentiation (58, 62), it would be critical to delineate if these cell types, which may occupy the same niches within the SLOs, act in concert to efficiently induce humoral responses.

While the migration of T cells from the T cell zone to the T-B border, and then to the follicle during Tfh differentiation, is well studied, there remains much to be understood about the spatio-temporal dynamics of this process with respect to T cell interactions with specific DC subsets. In other words, during the DC-phase of Tfh cell differentiation, do the differentiating T cells sequentially encounter different DC subsets? What specific signaling pathways and transcriptional programs are activated at each step of this process that then drives the next step of Tfh cell differentiation? Development of advanced microscopic techniques like multi-photon microscopy and multi-parameter immunofluorescence to simultaneously visualize T cells and antigen-bearing DC subsets would be required to address these questions. These techniques could be used in conjunction with next generation sequencing platforms such as single-cell RNA-Seq and spatial transcriptomics to identify the impact of different DC subsets on specific stages of Tfh cell differentiation.

Finally, Tfh cells can play either a beneficial or detrimental role in different diseases. First, Tfh cells are crucial in mediating protective antibody responses against pathogens as well as driving effective vaccine responses. However, we suggest that current vaccination strategies may be suboptimal in reaching DCs that are most efficient in priming Tfh responses (30). On the other hand, Tfh cells have been implicated in a diverse range of diseases such as allergy, autoimmunity, transplant rejection, and even cancer (107–112). Currently, little is known regarding the DC-Tfh axis in the context of disease. Work will be needed to determine how DCs impact disease initiation, progression, or severity by controlling the magnitude and/or type of Tfh response. Manipulation of DCs could potentially provide a therapeutic avenue to correct “misguided” or inadequate Tfh responses.

## Author contributions

JK, SE, and AW wrote the first draft of the manuscript. SA and UY wrote sections of the manuscript and contributed intellectual review. All authors contributed to manuscript revision, read, and approved the submitted version.

### Conflict of interest statement

JK is employed by the company AstraZeneca AB. The remaining authors declare that the research was conducted in the absence of any commercial or financial relationships that could be construed as a potential conflict of interest.
